# cPCET versus HAT: A Direct Theoretical Method for Distinguishing X–H Bond‐Activation Mechanisms

**DOI:** 10.1002/anie.201805511

**Published:** 2018-08-29

**Authors:** Johannes E. M. N. Klein, Gerald Knizia

**Affiliations:** ^1^ Molecular Inorganic Chemistry Stratingh Institute for Chemistry Faculty of Science and Engineering University of Groningen Nijenborgh 4 9747 AG Groningen The Netherlands; ^2^ Department of Chemistry Pennsylvania State University 401A Chemistry Bldg University Park PA 16802 USA

**Keywords:** computational chemistry, electron flow, hydrogen atom transfer, intrinsic bond orbitals, proton-coupled electron transfer

## Abstract

Proton‐coupled electron transfer (PCET) events play a key role in countless chemical transformations, but they come in many physical variants which are hard to distinguish experimentally. While present theoretical approaches to treat these events are mostly based on physical rate coefficient models of various complexity, it is now argued that it is both feasible and fruitful to directly analyze the electronic N‐electron wavefunctions of these processes along their intrinsic reaction coordinate (IRC). In particular, for model systems of lipoxygenase and the high‐valent oxoiron(IV) intermediate TauD‐J it is shown that by invoking the intrinsic bond orbital (IBO) representation of the wavefunction, the common boundary cases of hydrogen atom transfer (HAT) and concerted PCET (cPCET) can be directly and unambiguously distinguished in a straightforward manner.

The transfer of a net hydrogen atom as part of a chemical reaction can proceed in many different ways. Depending on the circumstances, viewing this process as the coupled but distinct transfer of a proton and an electron can be more appropriate than viewing it as transfer of an actual hydrogen atom. An umbrella term covering reactions of this type is proton‐coupled electron transfer (PCET).^1^ Such reactions are of broad relevance in contexts ranging from biological processes, such as some of the key steps related to the function of Photosystem II,^2^ to hydrocarbon combustion, in for example the engine of a car.^3^ Their fundamental understanding is therefore vital for future developments in the associated research fields.

The most fundamental scenarios to be distinguished are stepwise processes in which electron and proton transfer occur in individual steps, and concerted PCET (cPCET), where the proton and the electron are transferred simultaneously. Reactions in which electron and proton travel together as a true hydrogen atom will be called hydrogen atom transfer (HAT); the more general term cPCET will be used only when proton and electron are transferred in concert, but do not travel together, a definition similar to the one used by Shaik and co‐workers.7a Scheme 1 shows two representative cases, which we will discuss in detail below. Unfortunately, the use of these terms is far from consistent in the literature and at times much confusion can arise when these terms are used interchangeably.

**Scheme 1:**
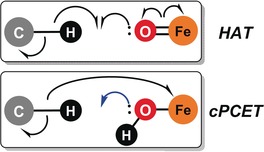
Representation of the electron flow in HAT and cPCET events from C(sp^3^)−H bonds to an acceptor (Fe^IV^=O or Fe^III^−OH). Electron flow for single‐electron events is depicted in black and blue for the movement of electron pairs.

Identifying which of the outlined mechanistic scenarios is operational can be challenging, as all scenarios involve the same net transfer of one electron and one proton and therefore cannot be distinguished by knowledge of properties of the reactants and products alone. Consequently, the concrete nature of the process effecting this transfer is a frequent matter of debate. Mechanistic insight is primarily gained by indirect inference, based on various physical models of the imagined sub‐processes of electron transfer (ET) and proton transfer (PT) and their coupling.^1^ In certain cases, directly accessible thermodynamic information on model compounds is sufficient to, for example, rule out stepwise processes.1e Frequently, however, complex physical models must be constructed to provide a basis for comparison to experimentally accessible information^1^ (for example, kinetic isotope effects or influences of substrate properties on rate behavior). These models have proven very successful in providing a detailed quantitative physical picture of PCET events in many contexts. For example, the Hammes–Schiffer group has employed quantitative and qualitative diagnostics based on 1) electronic transition/proton tunneling times;^4^ 2) the nonadiabatic coupling matrix element along the proton coordinate;^4^ 3) changes to the charge distribution using indicators such as dipole moment, electrostatic potential, or partial charges;^5^ and 4) changes in spin density.^6^ An alternative tool is, for example, the analysis of deformation energies, as proposed by the group of Shaik.^7^

Nevertheless, if we only pose the question of how the various PCET processes can be distinguished (for example, regarding sequentiality of electron and proton transfer), and which chemical bond transformations they are accompanied and influenced by, then the construction of such quantitative rate models may not be the most direct way to obtain this information. With modern software and computers it is absolutely possible to determine approximate but qualitatively correct (Kohn–Sham) electronic wave functions for most of the involved species and, based on those, also determine all likely intrinsic reaction paths for possible PCET events and compare their barriers. Once the most favorable reaction path has been determined, it should be possible to simply analyze the obtained trajectory of the ground state N‐electron wave function directly to clarify the concrete nature of the process. After all, the N‐electron wave function contains all information about the N‐electron system which is physically observable. Additionally, recently introduced analytic methods, such as the intrinsic bond orbital (IBO)^8^ transformation, provide an exact representation of any Kohn–Sham density functional theory (DFT) wavefunction, which is well amenable to the analysis of electronic structure changes in intuitive terms. We have previously demonstrated that the changes which IBOs undergo along a given reaction path can be linked to curly arrows^9^ and are indeed suitable for the investigation of C(sp^3^)−H activation reactions.^10^ These previously investigated reactions were of closed shell nature and only involved the movement of electron pairs. As a result, previous investigations did not give rise to the challenges that open shell systems, especially in homolytic bond cleavage, pose to most computational chemistry methods, and in particular to single‐reference methods such as DFT.^11^ Yet DFT does frequently allow for the qualitative, and even quantitative, description of complex chemical transformations (including reactions involving PCET)^12^ and its software implementations have by now reached a state of maturity allowing for in‐depth studies of large (and more importantly, experimentally accessible) systems. Analysis of stationary points for a cPCET reaction of an Fe^III^−OH complex with TEMPOH^13^ prompted us to explore the possibilities of monitoring electron flow in such PCET transformations using the IBO representation, to reveal their reaction mechanisms directly.

We initiated our studies by analyzing two reactions from the field of bioinorganic chemistry^14^ where C(sp^3^)−H bond oxidation occurs either via cPCET, following the above definition, or a HAT mechanism. For the cPCET case, we selected the well‐studied reaction of lipoxygenase,^15^ an Fe^III^−OH active site which breaks one of the C(sp^3^)−H bonds of arachidonic acid, and for HAT we selected the C(sp^3^)−H oxidation event from the oxoiron(IV) intermediate in taurine dioxygenase (TauD‐J),^16^ which oxidizes a C(sp^3^)−H bond of taurine. Structural depictions for the active sites and transition states for C−H bond activation are shown in Figure 1.

**Figure 1:**
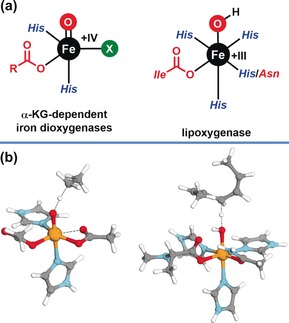
a) Lewis structure depictions of active sites for α‐KG‐dependent iron dioxygenases and lipoxygenase.^14^ b) Computed transition states for C−H bond oxidation by models for TauD‐J and lipoxygenase.

Based on these two reactions we will demonstrate that it is indeed possible to differentiate between cPCET and HAT in a straightforward and chemically intuitive way using IBOs. Our approach therefore provides a tool for unambiguous mechanism assignment with the potential for very broad applicability.

For the cPCET reaction of lipoxygenase, we used the model system previously employed by Soudackov and Hammes–Shiffer^6^ consisting of a high‐spin (*S*=5/2) Fe^III^−OH unit ligated by three imidazoles mimicking histidine residues, a carboxylate residue mimicking an isoleucine, and an amide mimicking an asparagine residue. The substrate was mimicked as a 1,4‐diene with a simple hydrocarbon framework (see Figure 1, bottom right). This reaction has not only been identified to follow a cPCET mechanism, but also provided a platform for the evaluation of theoretical methods.^3^, ^7^, ^17^

For the HAT reaction of the high‐valent oxoiron(IV) intermediate TauD‐J, we selected a model system studied previously by Ye and Neese.^18^ This consists of a high‐spin (*S*=2) Fe^IV^=O unit ligated by two imidazoles and one acetate mimicking a 2‐His‐1‐carboxylate facial triad^19^ and one additional acetate mimicking the coordination of decarboxylated α‐ketoglutarate. We note that several reaction channels have been discussed for oxoiron(IV) complexes for HAT reactions^20^ and we will focus on the σ‐pathway associated with the *S*=2 spin state which has been judged to be energetically most favorable in the present case by Ye and Neese.

We first computed the transition states for the C−H bond breaking events at the B3LYP^21^/def2‐SVP^22^ level of theory in the gas phase. We selected the B3LYP functional, as it has a proven track record for reactions of this type and provides satisfactory results even for challenging Fe‐based systems,^23^ despite its many known shortcomings. The def2‐SVP basis set is well balanced and has also been used successfully in several instances for related systems.^24^ To cross‐check, we also evaluated how the choice of functional and basis set affects our conclusions. All tested combinations produced consistent classification of cPCET vs. HAT and therefore these data are given in the Supporting Information, Figures S1 and S2.

We begin by demonstrating how IBOs can be used to identify HAT in the case of TauD‐J, which may be regarded as the simpler case. We begin our analysis by producing IBOs for the α and β spin manifold; next, we identify the localized orbitals of the C−H σ‐bond, and then follow the changes that they undergo along the IRC. In Figure 2 a we show how the α and β spin IBOs of the C−H bond evolve along the IRC. This in principle allows us to categorize C−H bond breaking reactions in a chemically intuitive way. As outlined above, if the C−H bond is broken via HAT, it would be expected that one of the localized IBOs would be transformed from a C−H σ‐bond into a part of the O−H bond in the present case. This is indeed what is observed: the IBO belonging to the α spin manifold becomes part of the newly formed O−H σ‐bond, whereas the IBO of the β spin manifold remains on the carbon atom of the substrate. This scenario is consistent with the expected σ‐pathway previously described for HAT reactions of oxoiron(IV) complexes. A high‐spin Fe^III^−OH intermediate is formed, which is antiferromagnetically coupled to the radical on the substrate.^20^ In short, we can see that the electron pair of the C−H bond in the substrate is cleaved homolytically, where one electron travels together with the proton and the second electron is left behind forming a substrate radical. As the proton and the electron are transferred together, the newly formed O−H bond should consist of one (here α) electron from the C(sp^3^)−H bond and one electron from the Fe^IV^=O unit. This does indeed happen and the O‐centered σ‐lone pair, which provides the β electron is shown in Figure 2 c.

**Figure 2:**
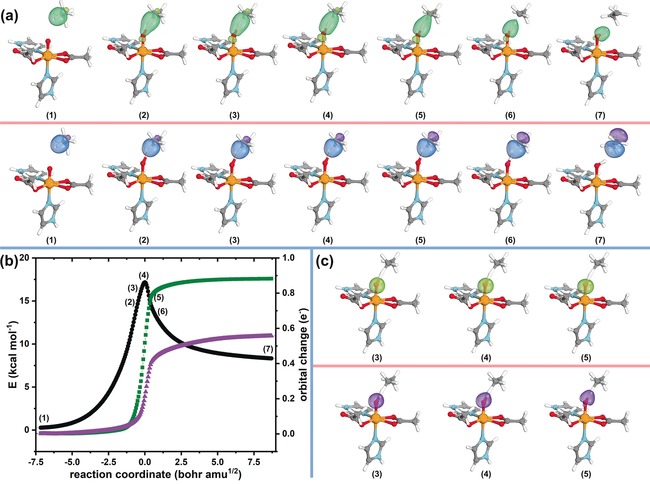
C−H activation by a small TauD‐J model complex. a) Changes of C−H IBO along IRC (α IBO green and β IBO purple). b) Plot of the IRC with energies shown in black circles (referenced to the fully optimized endpoint of the IRC), and IBO changes of the C−H bond along the IRC shown in green squares and purple triangles for the α and β IBOs, respectively. IBO changes are plotted as the root‐mean‐square deviation of the orbital partial charge distribution among the atoms with respect to the initial partial charge distribution. c) α and β IBOs of the O‐centered lone pair that provides one electron towards the newly formed O−H bond.

Thus far, this procedure would require a step by step analysis of every individual point of the IRC and then would require us to estimate by how much a given IBO has changed. In previous studies of closed‐shell reactions,^25^ including C(sp^3^)−H activation processes,^10^ we have simplified the process of identifying the IBOs which undergo changes by computing the root‐mean‐square deviation of every IBO from the initial partial charge distribution along the intrinsic reaction coordinate. A plot of these values (orbital change, plotted in units of e^−^) gives immediate insight into which IBOs are participating in bond making and bond breaking along the reaction path. IBOs not involved in this process do not undergo significant changes and in principle do not require inspection (we only show the changes to the C−H bonds in Figures 2 b and 3 b; all other changes are not shown for clarity). The corresponding plot for the HAT reaction studied here is shown in Figure 2 b. Furthermore, this plot clearly demonstrates that the electron flow associated with HAT is continuous and thus that our description truly captures how the reaction occurs.

For the second reaction we studied the cPCET reaction of lipoxygenase and followed the same procedure. First, we followed the changes of the α and ß electrons of the C(sp^3^)−H bond along the IRC (Figure 3 a). As outlined above, for a cPCET reaction we would expect the proton and the electron to not travel together, but rather take separate paths. In line with this anticipation, we do observe this very behavior along the reaction path of the lipoxygenase model with the 1,4‐diene model substrate. The α electron remains on the substrate along the entire IRC, whereas the ß electron is transferred to the iron center, rendering its transfer independent of the proton. This independent proton transfer is confirmed by inspecting the electron pairs on the oxygen of the Fe−OH unit. As expected, the proton is forming a new O−H bond with a lone pair on the oxygen atom, supporting the observation of proton transfer rather than hydrogen atom transfer (Figure 3 c). This behavior is characteristic for cPCET. This clean distinction between HAT and cPCET mechanisms demonstrates how powerful the analysis of the electron flow by IBOs can be, including both closed and open shell pathways. The plot shown in Figure 3 b shows that the electron flow for cPCET is also captured as a continuous process.

**Figure 3:**
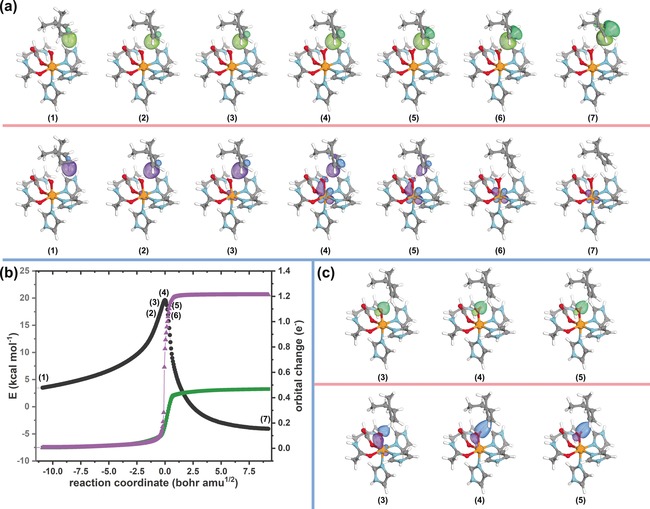
C−H activation by a lipoxygenase model complex. a) Changes of C−H IBO along IRC (α IBO green and β IBO purple). b) Plot of the IRC, with energies shown in black circles (referenced to the fully optimized endpoint of the IRC), and IBO changes of the C−H bond along the IRC shown in green squares and purple triangles for the α and β IBOs, respectively. IBO changes are plotted as the root mean square deviation of the orbital partial charge distribution among the atoms with respect to the initial partial charge distribution. c) IBOs of O‐centered α and β IBOs from O−H bond with the released proton.

In summary, we have used two prototypical model systems that cleave C(sp^3^)−H bonds to demonstrate that the electron flow of open shell reactions can be readily studied using IBOs. This simple and straightforward tool is apparently capable of differentiating electronic mechanisms even in challenging scenarios, such as occurring in HAT and cPCET reactions. We therefore believe this approach may shed light into many other challenging transformations.

## Conflict of interest

The authors declare no conflict of interest.

## Supporting information

As a service to our authors and readers, this journal provides supporting information supplied by the authors. Such materials are peer reviewed and may be re‐organized for online delivery, but are not copy‐edited or typeset. Technical support issues arising from supporting information (other than missing files) should be addressed to the authors.

Supplementary

## References

[1] For representative review articles see:

[1a] S. Hammes-Schiffer , J. Am. Chem. Soc. 2015, 137, 8860–8871; 26110700 10.1021/jacs.5b04087 PMC4601483

[1b] D. R. Weinberg , C. J. Gagliardi , J. F. Hull , C. F. Murphy , C. A. Kent , B. C. Westlake , A. Paul , D. H. Ess , D. G. McCafferty , T. J. Meyer , Chem. Rev. 2012, 112, 4016–4093; 22702235 10.1021/cr200177j

[1c] J. J. Warren , T. A. Tronic , J. M. Mayer , Chem. Rev. 2010, 110, 6961–7001; 20925411 10.1021/cr100085k PMC3006073

[1d] M. H. V. Huynh , T. J. Meyer , Chem. Rev. 2007, 107, 5004–5064; 17999556 10.1021/cr0500030 PMC3449329

[1e] J. M. Mayer , Annu. Rev. Phys. Chem. 2004, 55, 363–390. 15117257 10.1146/annurev.physchem.55.091602.094446

[2] T. J. Meyer , M. H. V. Huynh , H. H. Thorp , Angew. Chem. Int. Ed. 2007, 46, 5284–5304; 10.1002/anie.200600917 17604381

[3] J. M. Mayer , J. Phys. Chem. Lett. 2011, 2, 1481–1489. 21686056 10.1021/jz200021y PMC3115700

[4a] J. H. Skone , A. V. Soudackov , S. Hammes-Schiffer , J. Am. Chem. Soc. 2006, 128, 16655–16663; 17177415 10.1021/ja0656548

[4b] A. Sirjoosingh , S. Hammes-Schiffer , J. Chem. Theory Comput. 2011, 7, 2831–2841; see also 26605474 10.1021/ct200356b

[4c] J. M. Mayer , D. A. Hrovat , J. L. Thomas , W. T. Borden , J. Am. Chem. Soc. 2002, 124, 11142–11147; 12224962 10.1021/ja012732c

[4d] Y. Georgievskii , A. A. Stuchebrukhov , J. Chem. Phys. 2000, 113, 10438–10450.

[5] A. Sirjoosingh , S. Hammes-Schiffer , J. Phys. Chem. A 2011, 115, 2367–2377. 21351757 10.1021/jp111210c

[6] A. V. Soudackov , S. Hammes-Schiffer , J. Phys. Chem. Lett. 2014, 5, 3274–3278. 25258676 10.1021/jz501655v PMC4170820

[7a] D. Usharani , D. C. Lacy , A. S. Borovik , S. Shaik , J. Am. Chem. Soc. 2013, 135, 17090–17104; 24124906 10.1021/ja408073m PMC3876471

[7b] D. Usharani , D. Janardanan , C. Li , S. Shaik , Acc. Chem. Res. 2013, 46, 471–482. 23210564 10.1021/ar300204y

[8] G. Knizia , J. Chem. Theory Comput. 2013, 9, 4834–4843. 26583402 10.1021/ct400687b

[9] G. Knizia , J. E. M. N. Klein , Angew. Chem. Int. Ed. 2015, 54, 5518–5522; 10.1002/anie.201410637 25737294

[10] J. E. M. N. Klein , G. Knizia , L. Nunes dos Santos Comprido , J. Kästner , A. S. K. Hashmi , Chem. Eur. J. 2017, 23, 16097–16103. 28922498 10.1002/chem.201703815

[11a] A. J. Cohen , P. Mori-Sánchez , W. Yang , Science 2008, 321, 792–794; 18687952 10.1126/science.1158722

[11b] A. J. Cohen , P. Mori-Sánchez , W. Yang , Chem. Rev. 2012, 112, 289–320. 22191548 10.1021/cr200107z

[12] For representative examples, see also:

[12a] H. Schwarz , Chem. Phys. Lett. 2015, 629, 91–101;

[12b] H. Schwarz , S. Shaik , J. Li , J. Am. Chem. Soc. 2017, 139, 17201–17212; 29112810 10.1021/jacs.7b10139

[12c] H. Schwarz , P. González-Navarrete , J. Li , M. Schlangen , X. Sun , T. Weiske , S. Zhou , Organometallics 2017, 36, 8–17.

[13] W.-M. Ching , A. Zhou , J. E. M. N. Klein , R. Fan , G. Knizia , C. J. Cramer , Y. Guo , L. Que, Jr. , Inorg. Chem. 2017, 56, 11129–11140. 28858496 10.1021/acs.inorgchem.7b01459 PMC8991986

[14] X. Huang , J. T. Groves , J. Biol. Inorg. Chem. 2017, 22, 185–207. 27909920 10.1007/s00775-016-1414-3 PMC5350257

[15] M. J. Knapp , K. Rickert , J. P. Klinman , J. Am. Chem. Soc. 2002, 124, 3865–3874. 11942823 10.1021/ja012205t

[16a] C. Krebs , D. Galonić Fujimori , C. T. Walsh , J. M. Bollinger, Jr. , Acc. Chem. Res. 2007, 40, 484–492; 17542550 10.1021/ar700066p PMC3870002

[16b] S. Kal , L. Que, Jr. , J. Biol. Inorg. Chem. 2017, 22, 339–365; 28074299 10.1007/s00775-016-1431-2

[16c] RSC Metallobiology Series No. 3, 2-Oxoglutarate-Dependent Oxygenases, 1st ed. (Eds.: C. Schofield, R. Hausinger), Royal Society of Chemistry, Cambridge, 2015;

[16d] J. C. Price , E. W. Barr , T. E. Glass , C. Krebs , J. M. Bollinger, Jr. , J. Am. Chem. Soc. 2003, 125, 13008–13009; 14570457 10.1021/ja037400h

[16e] J. M. Bollinger , C. Krebs , J. Inorg. Biochem. 2006, 100, 586–605. 16513177 10.1016/j.jinorgbio.2006.01.022

[17a] S. Hammes-Schiffer , A. V. Soudackov , J. Phys. Chem. B 2008, 112, 14108–14123; see also 18842015 10.1021/jp805876e PMC2720037

[17b] E. Hatcher , A. V. Soudackov , S. Hammes-Schiffer , J. Am. Chem. Soc. 2004, 126, 5763–5775; 15125669 10.1021/ja039606o

[17c] E. Hatcher , A. V. Soudackov , S. Hammes-Schiffer , J. Am. Chem. Soc. 2007, 129, 187–196; 17199298 10.1021/ja0667211

[17d] A. K. Harshan , T. Yu , A. V. Soudackov , S. Hammes-Schiffer , J. Am. Chem. Soc. 2015, 137, 13545–13555; 26412613 10.1021/jacs.5b07327 PMC4629534

[17e] N. Lehnert , E. I. Solomon , J. Biol. Inorg. Chem. 2003, 8, 294–305. 12589565 10.1007/s00775-002-0415-6

[18] S. Ye , F. Neese , Proc. Natl. Acad. Sci. USA 2011, 108, 1228–1233. 21220293 10.1073/pnas.1008411108 PMC3029731

[19] K. Koehntop , J. Emerson , L. Que, Jr. , J. Biol. Inorg. Chem. 2005, 10, 87–93. 15739104 10.1007/s00775-005-0624-x

[20a] M. Srnec , S. D. Wong , E. I. Solomon , Dalton Trans. 2014, 43, 17567–17577; 24916844 10.1039/c4dt01366b PMC4229428

[20b] S. Shaik , H. Chen , D. Janardanan , Nat. Chem. 2011, 3, 19–27; 21160512 10.1038/nchem.943

[20c] S. Shaik , Int. J. Mass Spectrom. 2013, 354–355, 5–14.

[21a] A. D. Becke , J. Chem. Phys. 1993, 98, 5648–5652;

[21b] A. D. Becke , Phys. Rev. A 1988, 38, 3098–3100; 10.1103/physreva.38.3098 9900728

[21c] C. Lee , W. Yang , R. G. Parr , Phys. Rev. B 1988, 37, 785–789; 10.1103/physrevb.37.785 9944570

[21d] P. J. Stephens , F. J. Devlin , C. F. Chabalowski , M. J. Frisch , J. Phys. Chem. 1994, 98, 11623–11627.

[22] F. Weigend , R. Ahlrichs , Phys. Chem. Chem. Phys. 2005, 7, 3297–3305. 16240044 10.1039/b508541a

[23] A. Altun , J. Breidung , F. Neese , W. Thiel , J. Chem. Theory Comput. 2014, 10, 3807–3820. 26588526 10.1021/ct500522d

[24] For representative examples, see:

[24a] J. E. M. N. Klein , B. Dereli , L. Que, Jr. , C. J. Cramer , Chem. Commun. 2016, 52, 10509–10512; 10.1039/c6cc05395e 27489080

[24b] M. Srnec , S. D. Wong , M. L. Matthews , C. Krebs , J. M. Bollinger , E. I. Solomon , J. Am. Chem. Soc. 2016, 138, 5110–5122; 27021969 10.1021/jacs.6b01151 PMC4927264

[24c] M. Srnec , E. I. Solomon , J. Am. Chem. Soc. 2017, 139, 2396–2407. 28095695 10.1021/jacs.6b11995 PMC5310988

[25] L. Nunes Dos Santos Comprido , J. E. M. N. Klein , G. Knizia , J. Kästner , A. S. K. Hashmi , Chem. Eur. J. 2017, 23, 10901–10905. 28593697 10.1002/chem.201702023

