# Supplementary material for: cPCET versus HAT: A Direct Theoretical Method for Distinguishing X–H Bond‐Activation Mechanisms
Source: Angew Chem Int Ed Engl. 2018 Aug 29;57(37):11913–7. doi: 10.1002/anie.201805511 (PMC6175160; doi:10.1002/anie.201805511)
Supplement: Supplementary file 1 — Supplementary [file ANIE-57-11913-s001.pdf]

Angewandte Chemie
Eine Zeitschrift der Gesellschaft Deutscher Chemiker

Supporting Information

cPCET versus HAT: A Direct Theoretical Method for Distinguishing X–H Bond-Activation Mechanisms

Johannes E. M. N. Klein and Gerald Knizia**

anie_201805511_sm_miscellaneous_information.pdf

Table of contents

**1. Computational Details**	S2
**2. Energetics for Reaction Paths**	S3
**3. Comparison of IBOs at Transition States with Various DFT Functionals**	S4-S5
**4. Coordinates of Stationary Points**	S6-S11
**5. References**	S11-S12

1. Computational Details

Reaction paths were optimized using the electronic structure code Gaussian 09 rev. D.01.[1] The B3LYP functional[2] was used in combination with the def2-SVP basis set.[3] Frequency calculations were carried out in order to validate that either a local minimum (no imaginary frequency) or a transition state was reached (single imaginary frequency). Intrinsic reaction coordinate (IRC) calculations were carried out using the HPC algorithm[4] for up to 200 steps. All calculations employ an *ultrafine* grid. Start geometries for the transition states were taken from ref [5] for the lipoxygenase model and constructed according to ref [6] for the TauD-*J* model. High spin states were considered in both cases, $S = 5/2$ for the lipoxygenase model and $S = 2$ for the TauD-*J* model.

For analysis of the electron flow, electronic structures were recomputed along the IRCs using the electronic structure code ORCA 4.0.1.[7] The B3LYP/G functional and def2-SVP basis set were also used. Calculations were accelerated by the RIJCOSX[8] approach using Weigend's universal fitting basis sets.[9] Calculations use Grid5 and GridX5. Intrinsic bond orbitals (IBOs)[10] (*iboexp* = 2) were generated using IboView,[11] and used for the analysis of the electron flow.

IBOs were also computed for various combinations of functionals and basis sets at the transition states to evaluate the sensitivity to the choice of method (*vide infra*). These computations were carried out in ORCA 4.0.1 as outlined above. For this purpose, we tested the M06-L[12] and PW6B95[13] functionals (which have recently been shown to accurately predict spin ground states for Fe-based complexes[14]) in combination with the def2-SVP basis set. In combination with the B3LYP functional we tested def2-TZVP[3] as a larger triple-ζ basis set, and ma-SVP[15] to probe the effect of diffuse basis set augmentation. In all cases, we found that changes due to the choice of functional and/or basis set are insignificant, and, most importantly, all combinations produced consistent classification of cPCET vs. HAT.

2. Energetics for Reaction Paths

Table S1: Energetics for C-H bond activation for a TauD-*J* model.*

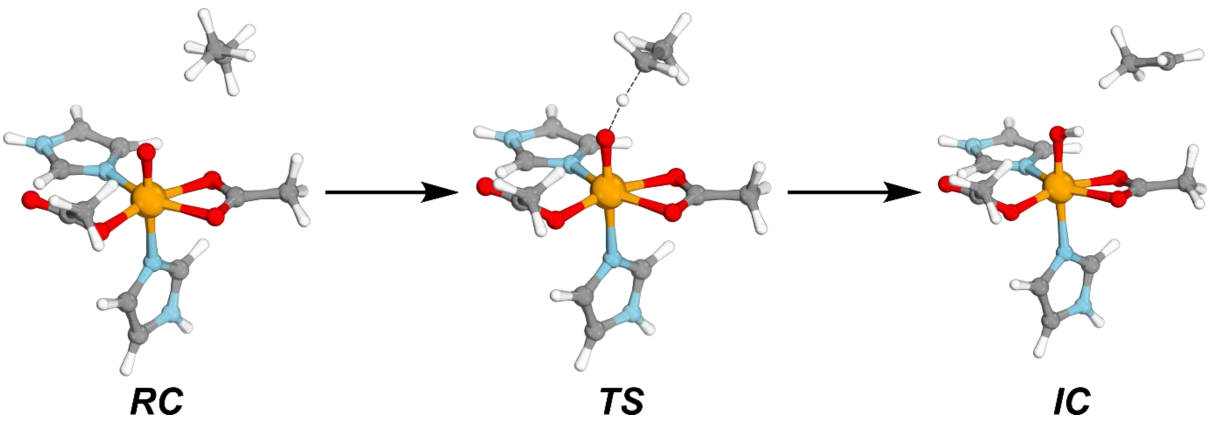

TauD-J	**ΔE (kcal mol^{-1})**	**ΔG$_{298}$ (kcal mol^{-1})**
RC	0.0	0.0
TS	17.1	13.8
IC	7.5	3.9

*Computed at the B3LYP/def2-SVP level of theory.

Table S2: Energetics for C-H bond activation for a lipoxygenase model.*

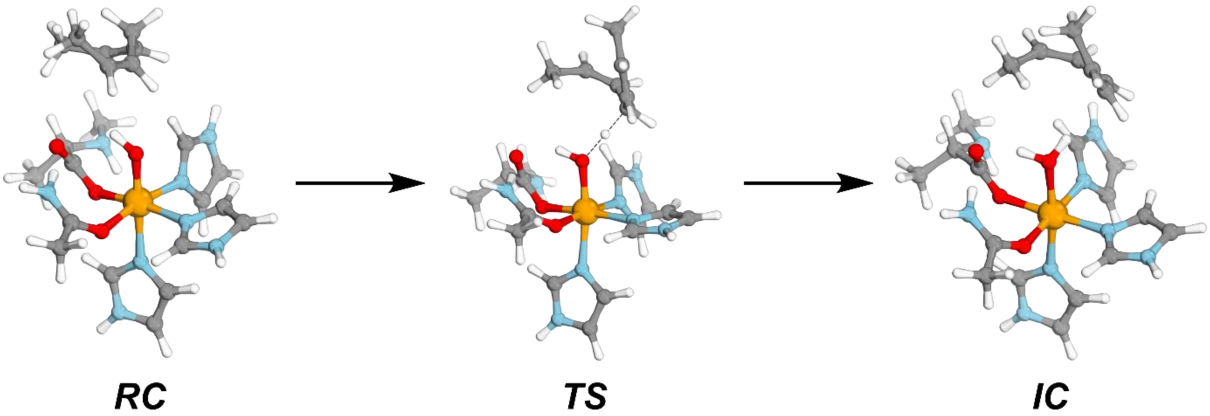

Lipoxygenase	**ΔE (kcal mol^{-1})**	**ΔG$_{298}$ (kcal mol^{-1})**
RC	0.0	0.0
TS	19.6	18.4
IC	-4.3	-5.3

*Computed at the B3LYP/def2-SVP level of theory.

3. Comparison of IBOs at Transition States with Various DFT Functionals

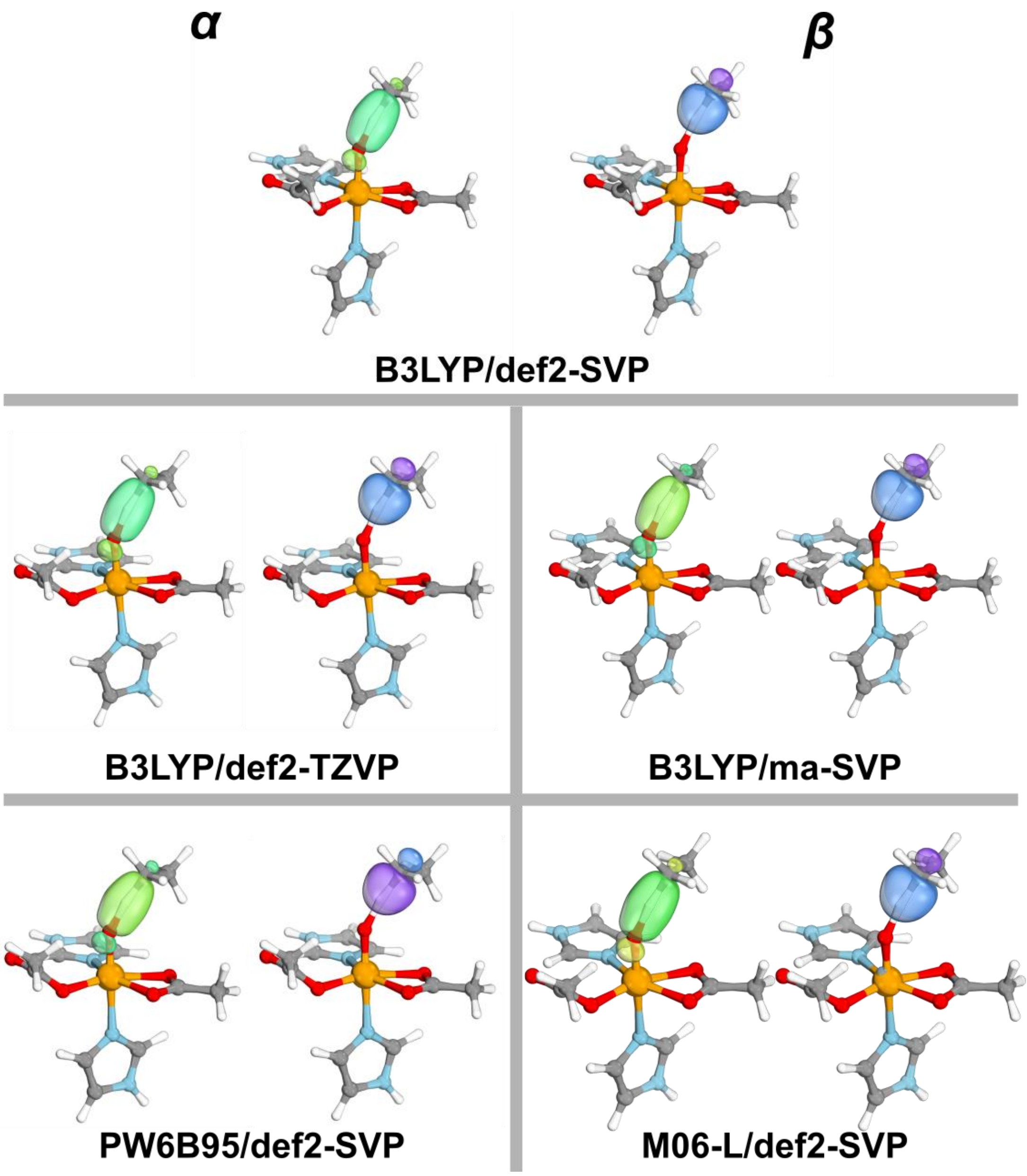

Figure S1: Comparison of the α and β IBOs involved in C-H bond breaking for the TauD-*J* model at the transition state using various DFT functionals and basis sets.

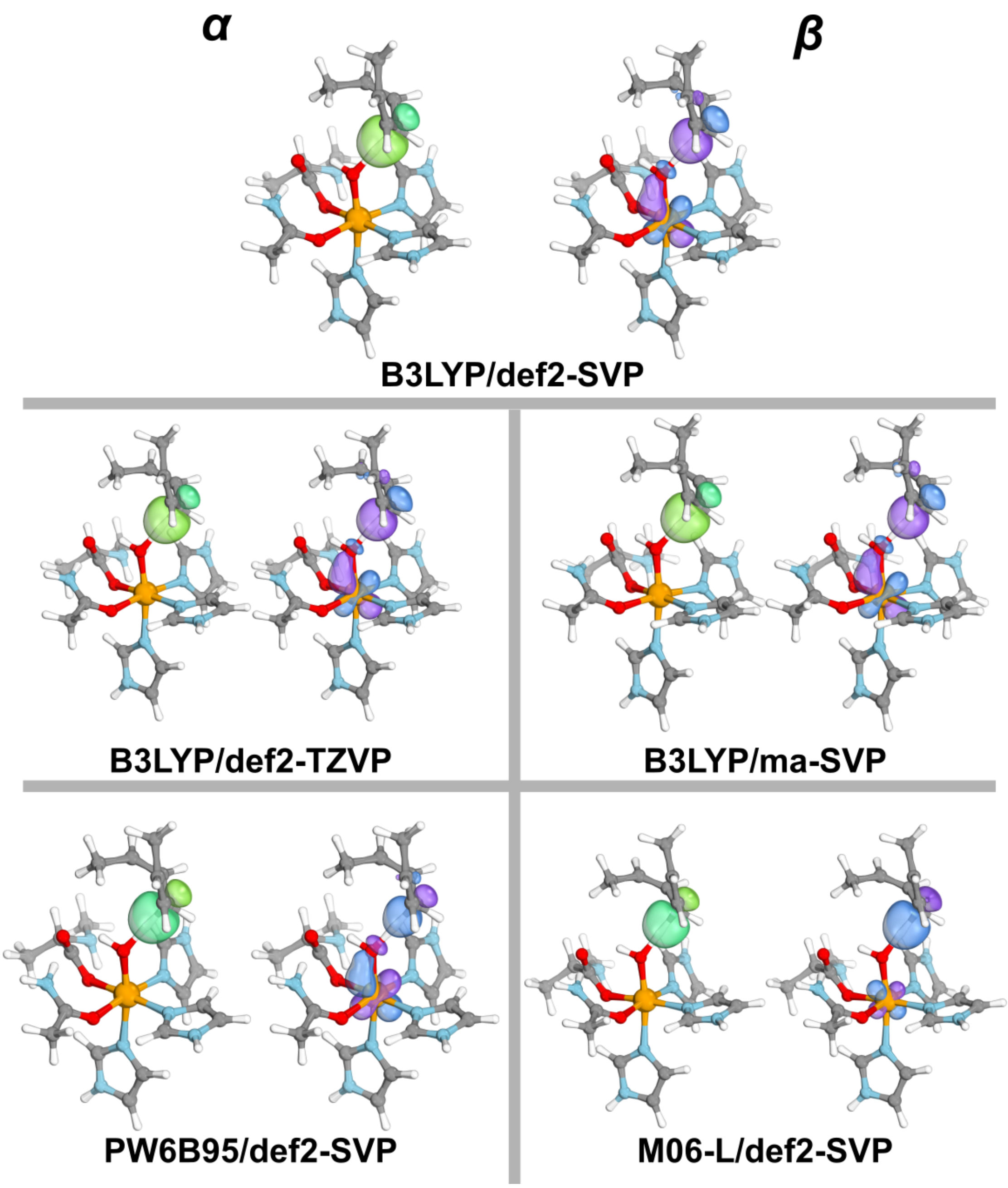

Figure S2: Comparison of the α and β IBOs involved in C-H bond breaking for the lipoxygenase model at the transition state using various DFT functionals and basis sets.

4. Coordinates of Stationary Points

Structures for the TauD-*J* model	
Fe -0.100060000 0.004847000 -0.572969000<br>N -0.039233000 -1.028601000 1.294313000<br>C 0.804943000 -0.834589000 2.362386000<br>H 1.518097000 -0.014246000 2.375028000<br>C 0.581263000 -1.823210000 3.288513000<br>H 1.034220000 -2.029070000 4.254287000<br>N -0.412134000 -2.616575000 2.748797000<br>H -0.807167000 -3.451156000 3.165608000<br>C -0.760796000 -2.109197000 1.542371000<br>H -1.492036000 -2.546638000 0.852286000<br>N -1.419251000 1.417815000 0.362963000<br>C -2.788358000 1.448935000 0.224050000<br>H -3.292915000 0.730927000 -0.415912000<br>C -3.294512000 2.467046000 0.992363000<br>H -4.309418000 2.820061000 1.152181000<br>N -2.201348000 3.049723000 1.602786000<br>H -2.218461000 3.835990000 2.241310000<br>C -1.091381000 2.388129000 1.197953000<br>H -0.077358000 2.622771000 1.513150000<br>O 1.482648000 1.391106000 0.176441000<br>C 1.424701000 1.959919000 -0.954422000<br>O 0.559054000 1.588073000 -1.795693000<br>C 2.402539000 3.052525000 -1.315463000<br>H 3.290582000 2.582193000 -1.768713000<br>H 1.964888000 3.737053000 -2.054381000<br>H 2.728524000 3.597861000 -0.419308000<br>O -1.711349000 -0.787287000 -1.231251000<br>C -1.859616000 -1.986457000 -1.761057000<br>O -2.146779000 -2.970585000 -1.096042000<br>C -1.651835000 -2.050330000 -3.259993000<br>H -1.939423000 -3.034511000 -3.651721000<br>O 0.909820000 -1.045239000 -1.256375000<br>H 3.476685000 -1.219534000 -0.927045000<br>C 4.519260000 -0.973465000 -0.670860000<br>H 5.119973000 -1.894193000 -0.760599000<br>C 4.611722000 -0.371439000 0.730550000<br>H 4.888361000 -0.268807000 -1.435437000<br>H 4.276146000 -1.090518000 1.496842000<br>H 3.967690000 0.518112000 0.816439000<br>H 5.643650000 -0.076248000 0.986521000<br>H -0.583153000 -1.866814000 -3.458096000<br>H -2.218301000 -1.251558000 -3.762242000	RC<br><br>E (Hartree) = -2327.35286247<br><br>G_{298} (Hartree) = -2327.090823
Fe 0.166353000 0.122841000 -0.475833000<br>N 0.113704000 -0.933831000 1.415156000<br>C 0.252879000 -0.433059000 2.688428000<br>H 0.324143000 0.637728000 2.864507000<br>C 0.292842000 -1.479581000 3.576570000<br>H 0.392880000 -1.512490000 4.657942000<br>N 0.177631000 -2.620549000 2.805947000<br>H 0.183164000 -3.575876000 3.142786000<br>C 0.073576000 -2.252541000 1.505885000<br>H 0.005676000 -2.936582000 0.650682000<br>N -2.058617000 0.437882000 -0.136233000<br>C -3.057709000 -0.314847000 -0.708024000<br>H -2.815837000 -1.118974000 -1.397869000<br>C -4.276673000 0.129444000 -0.257297000<br>H -5.292725000 -0.191564000 -0.469750000<br>N -3.993362000 1.169639000 0.605691000<br>H -4.668194000 1.725935000 1.116650000<br>C -2.645634000 1.320157000 0.649188000<br>H -2.126676000 2.064133000 1.250002000	TS<br><br>E (Hartree) = -2327.3255484<br><br>G_{298} (Hartree) = -2327.068843

O	0.282988000	2.072696000	0.670989000	
C	0.245406000	2.653620000	-0.454023000	
O	0.084395000	1.979489000	-1.510870000	
C	0.419219000	4.153271000	-0.549894000	
H	1.438746000	4.367301000	-0.909917000	
H	-0.281442000	4.571186000	-1.287137000	
H	0.283552000	4.629824000	0.429893000	
O	-0.409641000	-1.343316000	-1.574246000	
C	0.276019000	-2.388588000	-1.984187000	
O	0.357855000	-3.427332000	-1.343283000	
C	0.961649000	-2.208525000	-3.324453000	
H	1.412806000	-3.150473000	-3.662325000	
O	1.877033000	-0.107650000	-0.686933000	
H	3.022370000	0.291068000	-0.505760000	
C	4.125115000	0.848539000	-0.191675000	
H	4.869690000	0.049032000	-0.320838000	
C	3.957956000	1.362615000	1.215972000	
H	4.228138000	1.624361000	-0.964565000	
H	3.821710000	0.536531000	1.932049000	
H	3.078348000	2.020697000	1.297100000	
H	4.844781000	1.939420000	1.541404000	
H	1.741092000	-1.440000000	-3.198356000	
H	0.248188000	-1.831230000	-4.072962000	
Fe	0.035806000	-0.096108000	-0.489074000	IC<br><br>E (Hartree) = -2327.34097973<br><br>G_{298} (Hartree) = -2327.084687
N	-0.417340000	-0.945335000	1.424009000	
C	0.220020000	-0.737313000	2.626520000	
H	1.018790000	-0.005252000	2.716003000	
C	-0.317491000	-1.581989000	3.564448000	
H	-0.097209000	-1.733133000	4.617485000	
N	-1.289174000	-2.304489000	2.897333000	
H	-1.873154000	-3.032345000	3.291689000	
C	-1.321106000	-1.895777000	1.607869000	
H	-1.965263000	-2.306803000	0.819630000	
N	-1.346246000	1.648097000	0.005327000	
C	-2.637733000	1.800800000	-0.444405000	
H	-3.072088000	1.079089000	-1.130511000	
C	-3.182967000	2.930881000	0.113235000	
H	-4.160691000	3.393784000	0.011808000	
N	-2.191969000	3.460083000	0.916532000	
H	-2.259892000	4.300320000	1.478214000	
C	-1.104072000	2.656089000	0.822480000	
H	-0.166230000	2.820153000	1.348789000	
O	1.499596000	1.248041000	0.551498000	
C	1.838910000	1.675583000	-0.590417000	
O	1.202649000	1.290611000	-1.617238000	
C	3.023308000	2.597856000	-0.741731000	
H	3.928877000	1.972216000	-0.823345000	
H	2.934550000	3.202895000	-1.653921000	
H	3.135201000	3.236603000	0.145363000	
O	-1.522873000	-0.748875000	-1.430951000	
C	-1.835407000	-1.954284000	-1.845654000	
O	-2.374125000	-2.791798000	-1.134003000	
C	-1.466570000	-2.244001000	-3.288332000	
H	-1.902983000	-3.195158000	-3.619571000	
O	1.231579000	-1.437539000	-0.978288000	
H	1.818200000	-1.184201000	-1.705373000	
C	5.267088000	-0.579686000	-0.305038000	
H	5.267400000	-0.906605000	-1.349512000	
C	4.309747000	-1.160635000	0.674107000	
H	6.110879000	0.035916000	0.021869000	
H	3.320943000	-1.357217000	0.226108000	
H	4.164872000	-0.501557000	1.545992000	
H	4.673993000	-2.133219000	1.072658000	
H	-0.367224000	-2.303668000	-3.341300000	
H	-1.783813000	-1.420158000	-3.945199000	

Structures for the Lipoxygenase model	
Fe -0.908988000 -0.381088000 0.030173000<br>N -1.737273000 -2.093319000 -1.099258000<br>C -1.640782000 -2.320873000 -2.455581000<br>H -1.251509000 -1.569302000 -3.136082000<br>C -2.091158000 -3.585952000 -2.736849000<br>H -2.173151000 -4.136527000 -3.669923000<br>N -2.461969000 -4.122604000 -1.521950000<br>H -2.834491000 -5.053669000 -1.368854000<br>C -2.231376000 -3.198182000 -0.562624000<br>H -2.419086000 -3.345362000 0.497835000<br>N -3.042731000 0.226080000 0.471200000<br>C -4.241889000 -0.235916000 -0.029135000<br>H -4.286584000 -0.968900000 -0.830261000<br>C -5.279552000 0.371103000 0.634468000<br>H -6.357906000 0.281297000 0.535338000<br>N -4.684429000 1.209901000 1.551840000<br>H -5.164963000 1.813033000 2.210943000<br>C -3.342349000 1.095859000 1.421619000<br>H -2.612953000 1.645623000 2.011404000<br>N -1.008199000 0.832848000 -1.798263000<br>C 0.125367000 1.179097000 -2.390695000<br>H 1.106437000 0.790804000 -2.127205000<br>N -0.113070000 2.090819000 -3.356749000<br>H 0.593542000 2.524375000 -3.941301000<br>C -1.467529000 2.353021000 -3.382485000<br>H -1.915315000 3.055476000 -4.080141000<br>C -2.011996000 1.561019000 -2.403080000<br>H -3.051965000 1.470497000 -2.101341000<br>O -1.176701000 -1.622429000 1.726523000<br>C -0.458858000 -1.977999000 2.694798000<br>N 0.654134000 -1.353809000 3.049076000<br>H 1.005853000 -0.502591000 2.551469000<br>H 1.184129000 -1.690604000 3.843826000<br>C -0.888635000 -3.186224000 3.494472000<br>H -0.807747000 -4.081153000 2.857280000<br>H -0.287422000 -3.343403000 4.400189000<br>H -1.946091000 -3.073035000 3.774378000<br>O -0.475270000 1.299287000 1.065107000<br>C 0.683240000 1.713450000 1.460475000<br>O 1.562237000 0.981266000 1.933443000<br>C 0.920523000 3.222336000 1.336538000<br>H 2.004243000 3.374699000 1.461242000<br>C 0.189935000 3.961009000 2.475749000<br>H -0.901929000 3.836393000 2.385589000<br>H 0.503745000 3.573733000 3.458076000<br>H 0.413601000 5.038226000 2.458591000<br>N 0.563189000 3.629292000 -0.020462000<br>H -0.421490000 3.418724000 -0.180542000<br>C 0.877390000 5.001634000 -0.381458000<br>H 0.373574000 5.781267000 0.226589000<br>H 1.963613000 5.171773000 -0.296435000<br>H 0.602211000 5.176842000 -1.433567000<br>O 0.824419000 -0.862026000 -0.320077000<br>H 1.605982000 -0.495976000 0.126265000<br>H 5.293975000 -1.167593000 -2.455431000<br>C 4.905670000 -0.928564000 -1.446925000<br>H 5.788042000 -0.740705000 -0.818858000<br>C 4.128257000 -2.120983000 -0.933379000<br>H 3.135472000 -2.255203000 -1.379225000<br>C 4.512515000 -3.005805000 -0.000579000<br>H 3.804916000 -3.810229000 0.238863000<br>C 5.808380000 -3.053625000 0.756163000<br>H 6.363287000 -3.979930000 0.524244000<br>H 5.631086000 -3.066338000 1.845900000	RC<br><br>E (Hartree) = -2862.50224391<br><br>G_{298} (Hartree) = -2861.985759

H	6.472427000	-2.206655000	0.531968000
C	4.049710000	0.313581000	-1.553192000
H	3.398712000	0.353509000	-2.436747000
C	3.983206000	1.335548000	-0.685107000
H	3.276013000	2.144326000	-0.912321000
C	4.747691000	1.501449000	0.595349000
H	5.498210000	0.713992000	0.752545000
H	5.265611000	2.475706000	0.621764000
H	4.050013000	1.482030000	1.450204000

TS

E (Hartree) = -2862.47102281

G_{298} (Hartree) = -2861.956419

Fe	-0.680587000	-0.560704000	0.053766000
N	-0.307132000	-2.577425000	-0.789099000
C	0.157541000	-2.908615000	-2.045118000
H	0.287276000	-2.160957000	-2.822979000
C	0.401056000	-4.258439000	-2.100342000
H	0.769193000	-4.898207000	-2.897707000
N	0.075287000	-4.741030000	-0.850814000
H	0.142096000	-5.706556000	-0.547064000
C	-0.343541000	-3.701919000	-0.091822000
H	-0.662495000	-3.778708000	0.944863000
N	-2.888814000	-0.853926000	-0.120487000
C	-3.611854000	-1.888356000	-0.674425000
H	-3.128598000	-2.721367000	-1.178631000
C	-4.952824000	-1.671971000	-0.471552000
H	-5.832988000	-2.244536000	-0.751116000
N	-5.028847000	-0.482441000	0.220161000
H	-5.878795000	-0.025236000	0.531896000
C	-3.769269000	-0.023031000	0.410898000
H	-3.515699000	0.901599000	0.924200000
N	-0.575275000	0.357072000	-1.951321000
C	0.302217000	1.308232000	-2.230466000
H	1.136348000	1.609774000	-1.600897000
N	0.016492000	1.877591000	-3.421456000
H	0.527939000	2.645604000	-3.842067000
C	-1.110679000	1.269161000	-3.933123000
H	-1.546159000	1.550566000	-4.887981000
C	-1.468080000	0.325256000	-3.003264000
H	-2.307606000	-0.365181000	-3.024129000
O	-0.950891000	-1.592419000	1.950628000
C	-0.634923000	-1.398448000	3.143867000
N	-0.057271000	-0.282513000	3.583337000
H	0.195312000	0.501334000	2.950798000
H	0.173909000	-0.189163000	4.564504000
C	-0.909759000	-2.491059000	4.154234000
H	-0.242069000	-3.342732000	3.947346000
H	-0.757469000	-2.171928000	5.194464000
H	-1.943508000	-2.843479000	4.028152000
O	-1.198285000	1.348680000	0.747472000
C	-0.422893000	2.220282000	1.269543000
O	0.567894000	1.946352000	1.982259000
C	-0.765088000	3.689201000	0.995693000
H	0.152751000	4.263485000	1.201754000
C	-1.858175000	4.157085000	1.978357000
H	-2.796592000	3.601783000	1.813404000
H	-1.539788000	3.995844000	3.020346000
H	-2.070048000	5.230236000	1.858734000
N	-1.076092000	3.828679000	-0.423998000
H	-1.858871000	3.217216000	-0.652708000
C	-1.293173000	5.180920000	-0.906503000
H	-2.144116000	5.723387000	-0.444019000
H	-0.389315000	5.789918000	-0.735128000
H	-1.470692000	5.160191000	-1.993767000
O	1.214836000	-0.267648000	0.485835000
H	1.338221000	0.531206000	1.042735000
H	2.331806000	-0.350101000	-0.224799000
C	3.429462000	-0.435293000	-0.901132000

H	3.005970000	-0.923026000	-1.796492000	
C	4.198473000	-1.424431000	-0.117621000	
H	3.627724000	-2.346251000	0.054229000	
C	5.450543000	-1.417859000	0.400264000	
H	5.733646000	-2.324591000	0.950196000	
C	6.536385000	-0.392562000	0.296260000	
H	7.474874000	-0.873140000	-0.029078000	
H	6.752190000	0.054036000	1.284104000	
H	6.300458000	0.420436000	-0.400016000	
C	3.852318000	0.958418000	-1.189168000	
H	3.908007000	1.226654000	-2.252409000	
C	4.072660000	1.954013000	-0.301430000	
H	4.328953000	2.939453000	-0.709941000	
C	3.990832000	1.870317000	1.191882000	
H	3.867113000	0.839542000	1.551280000	
H	4.900832000	2.288358000	1.655454000	
H	3.141224000	2.465652000	1.570551000	
Fe	-0.923493000	-0.514416000	0.056665000	IC
N	-0.777847000	-2.626808000	-0.629804000	
C	-0.258489000	-3.106608000	-1.813809000	
H	0.075990000	-2.439238000	-2.604053000	E (Hartree) = -2862.50908703
C	-0.252730000	-4.479829000	-1.790199000	
H	0.071377000	-5.218301000	-2.518456000	
N	-0.779858000	-4.826468000	-0.565173000	G_{298} (Hartree) = -2861.994237
H	-0.915673000	-5.769695000	-0.218110000	
C	-1.080601000	-3.686028000	0.101982000	
H	-1.503576000	-3.649614000	1.103240000	
N	-3.107547000	-0.405307000	-0.243946000	
C	-3.992368000	-1.302352000	-0.801087000	
H	-3.659547000	-2.243522000	-1.232191000	
C	-5.270692000	-0.809481000	-0.702061000	
H	-6.231554000	-1.208190000	-1.015909000	
N	-5.142861000	0.409358000	-0.071305000	
H	-5.898586000	1.045292000	0.158761000	
C	-3.828846000	0.614903000	0.187427000	
H	-3.422067000	1.499015000	0.674042000	
N	-0.476953000	0.301191000	-1.966897000	
C	0.501786000	1.156210000	-2.214020000	
H	1.286569000	1.449753000	-1.521549000	
N	0.390828000	1.654720000	-3.466035000	
H	1.007023000	2.345278000	-3.880362000	
C	-0.723101000	1.094992000	-4.055787000	
H	-1.032451000	1.335142000	-5.069315000	
C	-1.252621000	0.256196000	-3.106916000	
H	-2.141584000	-0.366495000	-3.172238000	
O	-1.315548000	-1.358589000	2.021768000	
C	-1.044960000	-1.146072000	3.220154000	
N	-0.270144000	-0.142603000	3.634340000	
H	0.172953000	0.518515000	2.971922000	
H	-0.084182000	-0.020891000	4.621780000	
C	-1.607230000	-2.075907000	4.273624000	
H	-1.146304000	-3.069211000	4.153524000	
H	-1.432877000	-1.729775000	5.301689000	
H	-2.687761000	-2.192272000	4.106218000	
O	-1.007787000	1.534319000	0.689787000	
C	-0.077736000	2.231843000	1.196280000	
O	0.880548000	1.760206000	1.866404000	
C	-0.143026000	3.745814000	0.965247000	
H	0.853102000	4.144792000	1.215563000	
C	-1.176150000	4.379720000	1.917513000	
H	-2.188125000	3.996804000	1.705682000	
H	-0.933447000	4.148910000	2.967057000	
H	-1.194621000	5.475289000	1.815850000	
N	-0.369584000	3.963823000	-0.462170000	
H	-1.246150000	3.515492000	-0.726892000	

C	-0.314898000	5.341102000	-0.920040000
H	-1.064081000	6.024937000	-0.469230000
H	0.680802000	5.767031000	-0.709696000
H	-0.458418000	5.372343000	-2.012035000
O	1.193598000	-0.502630000	0.551433000
H	1.332293000	0.315963000	1.095087000
H	1.986640000	-0.654783000	0.011985000
C	3.954539000	-0.845699000	-1.275070000
H	3.409311000	-1.196525000	-2.161406000
C	4.325323000	-1.896836000	-0.382057000
H	3.718783000	-2.804995000	-0.496006000
C	5.374603000	-2.017074000	0.497582000
H	5.425597000	-2.962779000	1.050442000
C	6.526904000	-1.090625000	0.714093000
H	7.473389000	-1.636282000	0.552158000
H	6.564482000	-0.728046000	1.756646000
H	6.511371000	-0.220507000	0.046073000
C	4.204755000	0.567879000	-1.273444000
H	4.167825000	1.018435000	-2.274587000
C	4.370084000	1.460144000	-0.245577000
H	4.528407000	2.504832000	-0.539532000
C	4.266823000	1.224004000	1.227509000
H	4.076805000	0.173536000	1.482253000
H	5.187230000	1.542850000	1.746793000
H	3.444925000	1.832166000	1.645229000

5. References

[1] M. J. Frisch, G. W. Trucks, H. B. Schlegel, G. E. Scuseria, M. A. Robb, J. R. Cheeseman, G. Scalmani, V. Barone, B. Mennucci, G. A. Petersson, H. Nakatsuji, M. Caricato, X. Li, H. P. Hratchian, A. F. Izmaylov, J. Bloino, G. Zheng, J. L. Sonnenberg, M. Hada, M. Ehara, K. Toyota, R. Fukuda, J. Hasegawa, M. Ishida, T. Nakajima, Y. Honda, O. Kitao, H. Nakai, T. Vreven, J. A. Montgomery Jr., J. E. Peralta, F. Ogliaro, M. J. Bearpark, J. Heyd, E. N. Brothers, K. N. Kudin, V. N. Staroverov, R. Kobayashi, J. Normand, K. Raghavachari, A. P. Rendell, J. C. Burant, S. S. Iyengar, J. Tomasi, M. Cossi, N. Rega, N. J. Millam, M. Klene, J. E. Knox, J. B. Cross, V. Bakken, C. Adamo, J. Jaramillo, R. Gomperts, R. E. Stratmann, O. Yazyev, A. J. Austin, R. Cammi, C. Pomelli, J. W. Ochterski, R. L. Martin, K. Morokuma, V. G. Zakrzewski, G. A. Voth, P. Salvador, J. J. Dannenberg, S. Dapprich, A. D. Daniels, Ö. Farkas, J. B. Foresman, J. V. Ortiz, J. Cioslowski, D. J. Fox, *Gaussian 09*, Gaussian, Inc., Wallingford, CT, USA, **2009**.

[2] (a) A. D. Becke, *J. Chem. Phys.* **1993**, *98*, 5648-5652; (b) A. D. Becke, *Phys. Rev. A* **1988**, *38*, 3098-3100; (c) C. Lee, W. Yang, R. G. Parr, *Phys. Rev. B* **1988**, *37*, 785-789; (d) P. J. Stephens, F. J. Devlin, C. F. Chabalowski, M. J. Frisch, *J. Phys. Chem.* **1994**, *98*, 11623-11627.

[3] F. Weigend, R. Ahlrichs, *Phys. Chem. Chem. Phys.* **2005**, *7*, 3297-3305.

[4] (a) H. P. Hratchian, H. B. Schlegel, *J. Chem. Phys.* **2004**, *120*, 9918-9924; (b) H. P. Hratchian, H. B. Schlegel, in *Theory and Applications of Computational Chemistry* (Eds.: G. Frenking, K. S. Kim, G. E. Scuseria), Elsevier, Amsterdam, **2005**, pp. 195-249; (c) H. P. Hratchian, H. B. Schlegel, *J. Chem. Theory Comput.* **2005**, *1*, 61-69.

[5] A. V. Soudackov, S. Hammes-Schiffer, *J. Phys. Chem. Lett.* **2014**, *5*, 3274-3278.

[6] S. Ye, F. Neese, *Proc. Natl. Acad. Sci. U.S.A.* **2011**, *108*, 1228-1233.

[7] (a) F. Neese, *WIREs Comput. Mol. Sci.* **2012**, *2*, 73-78; (b) F. Neese, *WIREs Comput. Mol. Sci.* **2018**, *8*, e1327.
[8] (a) F. Neese, F. Wennmohs, A. Hansen, U. Becker, *Chem. Phys.* **2009**, *356*, 98-109; (b) F. Neese, *J. Comput. Chem.* **2003**, *24*, 1740-1747.
[9] F. Weigend, *Phys. Chem. Chem. Phys.* **2006**, *8*, 1057-1065.
[10] G. Knizia, *J. Chem. Theory Comput.* **2013**, *9*, 4834-4843.
[11] (a) G. Knizia, J. E. M. N. Klein, *Angew. Chem. Int. Ed.* **2015**, *54*, 5518-5522; (b) G. Knizia, http://www.iboview.org/.
[12] Y. Zhao, D. G. Truhlar, *J. Chem. Phys.* **2006**, *125*, 194101.
[13] Y. Zhao, D. G. Truhlar, *J. Phys. Chem. A* **2005**, *109*, 5656-5667.
[14] P. Verma, Z. Varga, J. E. M. N. Klein, C. J. Cramer, L. Que Jr, D. G. Truhlar, *Phys. Chem. Chem. Phys.* **2017**, *19*, 13049-13069.
[15] J. Zheng, X. Xu, D. G. Truhlar, *Theor. Chem. Acc.* **2011**, *128*, 295-305.
